# Astronomical and Hydrological Perspective of Mountain Impacts on the Asian Summer Monsoon

**DOI:** 10.1038/srep17586

**Published:** 2015-12-01

**Authors:** Bian He, Guoxiong Wu, Yimin Liu, Qing Bao

**Affiliations:** 1 State Key Laboratory of Numerical Modeling for Atmospheric Sciences and Geophysical Fluid Dynamics, Institute of Atmospheric Physics, Chinese Academy of Sciences, Beijing 100029.

## Abstract

The Asian summer monsoon has great socioeconomic impacts. Understanding how the huge Tibetan and Iranian Plateaus affect the Asian summer monsoon is of great scientific value and has far-reaching significance for sustainable global development. One hypothesis considers the plateaus to be a shield for monsoon development in India by blocking cold-dry northerly intrusion into the tropics. Based on astronomical radiation analysis and numerical modeling, here we show that in winter the plateaus cannot block such a northerly intrusion; while in summer the daily solar radiation at the top of the atmosphere and at the surface, and the surface potential temperature to the north of the Tibetan Plateau, are higher than their counterparts to its south, and such plateau shielding is not needed. By virtue of hydrological analysis, we show that the high energy near the surface required for continental monsoon development is maintained mainly by high water vapor content. Results based on potential vorticity–potential temperature diagnosis further demonstrate that it is the pumping of water vapor from sea to land due to the thermal effects of the plateaus that breeds the Asian continental monsoon.

The precipitation associated with the Asian summer monsoon (ASM) covers a vast area^1^,^2^ and is important to the regional economy and culture. However, its anomalies usually bring drought or flood and can cause serious damage. Revealing the driving mechanism of its formation and variation has long been the aim of both paleoclimate and modern climate studies because such knowledge can contribute to the improvement of weather forecasts and climate prediction and projection, and to sustainable social development. The onset and maintenance of the ASM are highly related to the Tibetan Plateau (TP)^3^,^4^,^5^,^6^,^7^,^8^ and Iranian Plateau (IP) topography (TIP)^1^,^9^,^10^, and their thermal forcing and mechanical forcing are considered the main driving mechanism of the ASM besides the land-sea thermal contrast. The thermal forcing hypothesis suggests that^8^,^9^,^10^,^11^,^12^ since more than 85% of atmospheric water vapor content is confined to a boundary layer below 3 km, a mechanism is required to transport this water vapor from the surface to the free atmosphere to form monsoonal clouds. The surface sensible heating of the TIP can pump the low-layer moist air upward to higher layers much like a sensible-heat-driven air-pump (SHAP), forming the precipitation of the East ASM (EASM) and the northern branch of the South ASM (SASM)^10^. However, how the TIP-SHAP can transport water vapor horizontally from sea to land to support the continental monsoon is still unrevealed. The mechanical forcing hypothesis focuses on the formation of the SASM^13^,^14^: the Himalayas, the southern border of the TP, prevent the intrusion of cold and dry extra tropical air into North India, where the local subcloud moist entropy or equivalent potential temperature 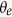 is large and monsoon convection can develop from a local instability. This hypothesis is facing challenges from both observations and numerical experiments: in winter there is cold/dry northerly invasion into India despite the existence of the TIP, and in summer there is no cold/dry equatorward flow in other global monsoon regions where no huge mountain ranges exist^15^,^16^. Furthermore, although both hypotheses emphasize the importance of high surface energy in producing monsoon convection, the relative contributions of water vapor and temperature to this surface energy and the roles played by the TIP have not been discussed. Most of these studies have been mainly confined to a meteorological framework^17^,^18^,^19^, and they have left several critical unsolved problems: whether such a TIP mechanical blocking mechanism exists for local monsoon development over North India? How the high surface energy in the continental monsoon area is produced, is it due to high surface temperature, or high surface water vapor content, and how and why remote TIP thermal forcing can help the development of the ASM over continental regions? This study tries to shed new light on these fundamental issues by employing astronomical, hydrological, and dynamical approaches.

Monsoon convection usually develops over a region where 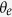 is high^20^. Because 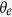 is a function of temperature *T* and moisture *q*, convection can develop over the tropical ocean away from the equator where surface temperature *T*_*sur*_ is high^21^. Tropical continents usually possess high *T*_*sur*_, which is necessary but not sufficient for convection to develop. *T*_*sur*_ over the Saudi Arabian Peninsula is very high, but there is no monsoon there. Thus, the fundamental question concerning the formation of the continental monsoon is how a high 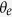 over land is generated^22^. By analyzing the solar radiation at the top of the atmosphere (TOA) and the downward shortwave radiation at the surface, as well as by using numerical modeling, we demonstrate here that the TIP cannot shield the tropics from cold and dry northerly intrusion whenever such cold/dry northerly advection exists; and in summer, when the daily solar radiation at the TOA and at the surface, and the surface potential temperature to the north of the TIP, are higher than to its south, there is no cold/dry northerly advection into India and the plateau shielding is not needed. With hydrological diagnosis we show that near the surface, moisture *q*_*sur*_ is more significant than potential temperature 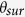 in producing high 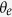; thus water vapor transport from the sea becomes crucial for continental monsoon formation. Furthermore, from the potential vorticity–potential temperature 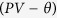 perspective, we show that the high 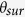 over the TIP, which is produced by local strong surface sensible heating, can generate a huge near-surface cyclonic circulation, and it is because of this unique cyclonic circulation that water vapor can be transported from sea to land and gives rise to the continental monsoon over North India and East Asia.

## Astronomical Perspective: no northerly intrusion into Indian monsoon area in summer

**Figure 1:**
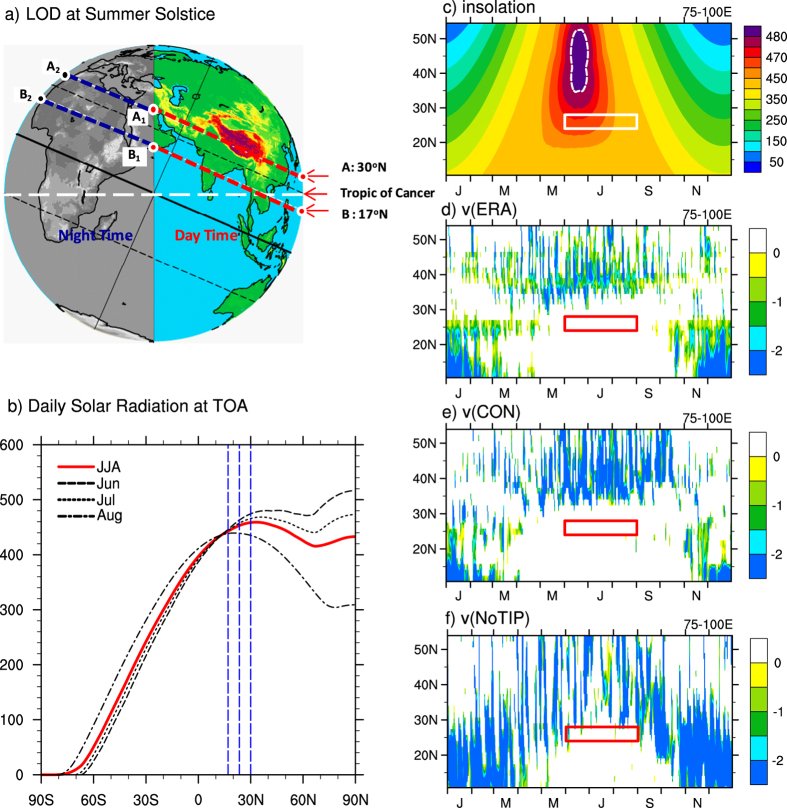
(**a**) Intensity of solar radiation (SR) and the length of day LOD at the TOA and at summer solstice; (**b**) latitude distributions of DSR in summer months; and annual evolution of (**c**) DSR (white-dashed denotes 480-contour), and of surface northerly occurrence along 75–100^o^E from (**d**) ERA-interim in 2001, (**e**) CON, and (**f**) NoTIP. The square in (**c–f**) indicates the SASM region (24–28^o^N) during JJA. Unit is (W m^−2^) in (**b**,**c**), and (m s^−1^) in (**d–f**). The three dashed blue lines in (**b**) indicate the latitudes of 30^o^N, 23.5^o^N, and 17^o^N, respectively. This figure was generated using NCAR Command Language (NCL) version 6.3.0, open source software free to public developed by UCAR/NCAR/CISL/TDD, http://dx.doi.org/10.5065/D6WD3XH5, and Fig. 1a was also modified by licensed Microsoft Powerpoint and licensed Illustrator.

At the boreal summer solstice, the solar zenith angle at noon is zero along latitude 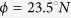. At this time, 6.5 degrees away from the Tropic of Cancer and at the top of the atmosphere, the solar radiation (SR) at latitude A (30^o^ N, where Shanghai is located) in the subtropics is the same as that at latitude B (17^o^ N, where Hyderabad is located) in the tropics (Fig. 1a). However, due to the tilting of the Earth’s axis, the length of day (LOD) in boreal summer increases with latitude, and the LOD at A (AA_1_/AA_2_ × 24 hours) is about one hour longer than at B (BB_1_/BB_2_ × 24 hours). Thus the daily SR (DSR) at 30^o^ N is 476 W m^−2^, about 6% more than that (449 W m^−2^) at 17^o^ N (Methods). Climate mean data from the Clouds and the Earth’s Radiant Energy System (CERES)^23^ also show that in June, July, and August the DSR is, respectively, 474, 466, and 436 W m^−2^ at 30^o^ N and 444, 443, and 438 W m^−2^ at 17^o^ N (Fig. 1b); and the JJA mean is 458 W m^−2^ at 30^o^ N but 442 W m^−2^ at 17^o^ N. These data indicate that in boreal summer the subtropical region receives more solar radiation than the tropical region. Diagnosis of the first year of CERES daily data shows that in winter the DSR is high in the tropics and low in the high latitudes, whereas in summer it is higher in the extratropics than in the tropics, with a maximum of more than 480 W m^−2^ located near 45^o^ N (Fig. 1c). Analysis of the daily occurrence of a northerly at the near-surface level (σ = 0.99) in the same year and based on ERA-interim data^24^ demonstrates that across the Indian monsoon domain of 75–100^o^E, cold/dry northerly advection events exist from the subtropics to the tropics in the winter half-year despite the TIP mountain barrier (Fig. 1d). On the contrary, in summer there is no northerly occurrence in this SASM region. This may be due to the fact that the summertime DSR is higher in the extratropics than in the tropics, as shown in Fig. 1c.

To verify the above inference, numerical experiments are performed based on the Spectral Atmospheric Model of LASG/IAP (SAMIL) (Methods). The observed seasonally varying SST is used to prescribe the lower boundary condition. The DSR in these experiments is the same as that shown in Fig. 1c. The near-surface moist entropy, measured in terms of equivalent potential temperature


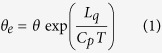


and precipitation, are diagnosed from the experiment outputs and compared to their counterparts in ERA40^25^, where 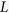, *C*_*p*_, and *q* denote, respectively, latent heat of vaporization, specific heat at constant atmospheric pressure, and specific humidity. The first experiment is simply the SAMIL climate integration, defined as the CON run. The second experiment, named NoTIP, is the same as CON except the TIP is removed (Methods). The daily outputs from these experiments show similar features to those observed: although in winter there is strong northerly advection into the tropics, in summer there is hardly any northerly intrusion from the subtropics into the tropical monsoon region even when the TIP is removed in the NoTIP run (Fig. 1e,f).

**Figure 2:**
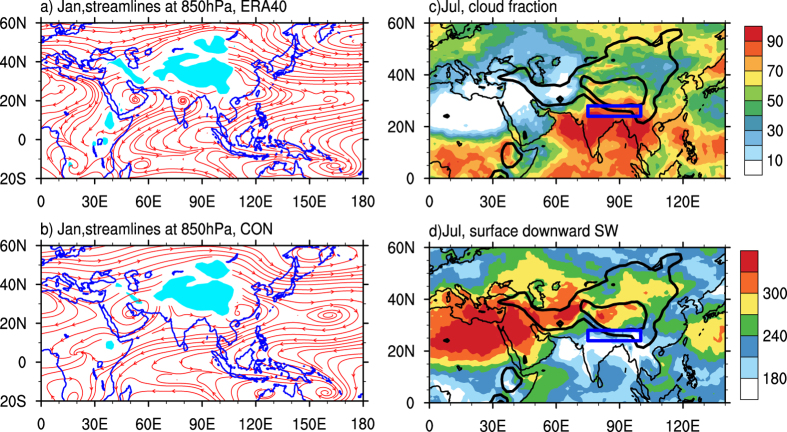
Climatological mean January streamfield at 850 hPa produced from (a) ERA40 and (b) the CON experiment; and the monthly mean for July 2001 of (c) cloud fraction (%) and (d) downward shortwave radiation at the surface (W m^−2^) produced from CERES. This figure was generated using NCL version 6.3.0: http://dx.doi.org/10.5065/D6WD3XH5.

In winter, cold air flow certainly cannot climb over the massive, high TIP to reach India. How, then, can the cold/dry northerly advection invade India from the subtropics to the tropics in the winter half-year despite the TIP mountain barrier, as shown in Fig. 1d? By diagnosing the ERA40 reanalysis we found that this is because the frequent cold/dry northwesterlies or northeasterlies in the lower troposphere can go around the southern rim of the giant TIP and invade India (Fig. 2a), implying that the TIP cannot protect India from northerly intrusion in winter. This feature is well simulated in the CON run, as shown in Fig. 2b.

The seasonal variation of the DSR at the TOA makes the subtropical continent become the atmospheric heat sink in winter and the source in summer. The continental heating in summer produces a continental-scale cyclonic circulation near the surface and forms a moist/monsoon-type climate over the eastern continent and a dry/desert-type over its west^15^,^16^. The DSR at the TOA can also affect the surface wind in the monsoon region. This is because the surface wind is basically determined by the surface pressure gradient, which is closely related to the surface temperature gradient. Since the surface temperature is significantly influenced by the solar radiation reaching the earth’s surface and the surface characteristics, and since the surface downward shortwave radiation is determined by the DSR and local cloudiness, both the DSR and local cloudiness can significantly affect the surface wind distribution. Figure 2c,d presents respectively the distributions of cloudiness and downward shortwave radiation at the surface in July 2001. It shows that in the SASM sector of 75–100^o^E, there is more cloud (>80%) to the south of the TIP and less (<50%) to its north (Fig. 2c). Consequently the downward shortwave radiation at the surface is low (<210 W m^−2^) to the south of the TIP and high (>270 W m^−2^) to its north (Fig. 2d). This will then influence the distributions of surface potential temperature and wind in the ASM area.

**Figure 3:**
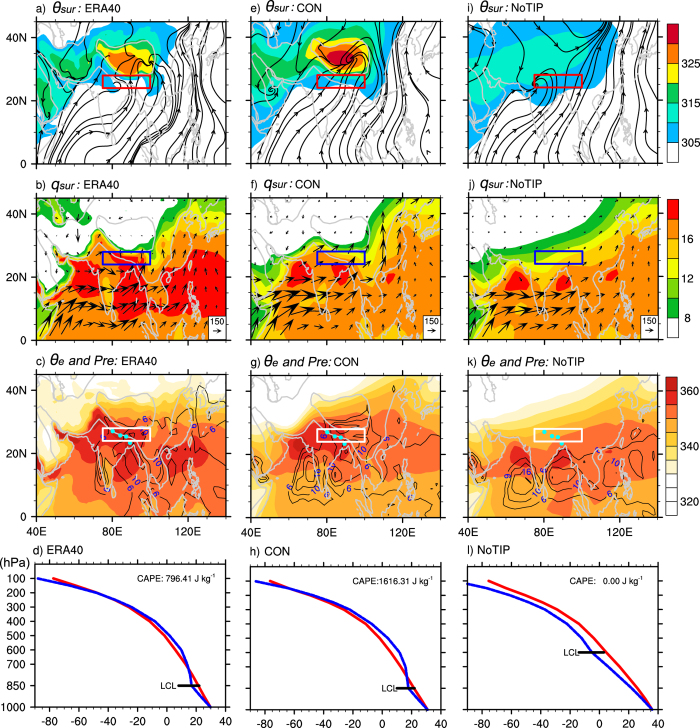
JJA-means of *θ*_*sur*_ (K) and streamfield (top row), *q*_*sur*_ (g kg^−1^) and 850-hPa water vapor fluxes (vectors, kg m^−1^s^−1^) (second row), and *θ* (K) and precipitation (contours, mm d-1) (third row); and of profiles of environmental temperature (K, red) and rising air-parcel temperature (K, blue) (bottom row) averaged over the four pale blue grid-points over North India shown in the third row, calculated from ERA-40 (a–d), CON (e–h), and NoTIP (i–l). The square indicates the SASM region of (24–28°N, 75–100°E). This figure was generated using NCL version 6.3.0: http://dx.doi.org/10.5065/D6WD3XH5.

The JJA mean potential temperature and streamline at the near-surface level σ = 0.99 in ERA40, CON, and NoTIP are presented in the top row of Fig. 3. The maximum surface potential temperature 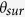 is over the TIP in ERA40 and CON (Fig. 3a,e). In NoTIP such a center over the TIP region disappears due to the lowered elevation in the region, and the maximum surface potential temperature 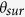 is located over the subtropics to midlatitudes (Fig. 3i), where both the DSR and downward shortwave radiation are high (Figs 1c and 2d). In all cases the surface flows converge toward the warm 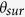 area. Consequently, a mean surface southerly prevails over the North Indian monsoon region, which can explain why there is hardly any transient northerly intrusion into India in summer. It is important to note that in other subtropical continental monsoon regions where no huge mountain ranges exist, the observed summer monsoon–type climate presents over the eastern continent where poleward flow dominates^15^,^16^. This then justifies the NoTIP results that even when there is no TIP, the southerly can dominate the South Asian monsoon region, and there should be no frequent cold/dry northerly advection there.

**Figure 4:**
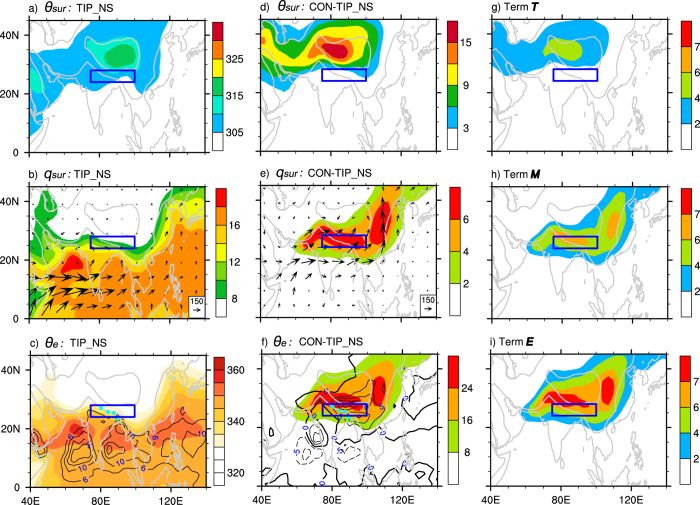
(**a–c**) and (**d–f**) are the same as Fig. 3(a–c) but, respectively, for TIP_NS and (CON-TIP_NS); and (**g–i**) the JJA-mean distributions of the ***T***, ***M***, and ***E*** terms in Formula (3). The square indicates the SASM region of (24–28°N, 75–100°E). This figure was generated using NCL version 6.3.0: http://dx.doi.org/10.5065/D6WD3XH5.

In boreal summer (JJA), the observed climate-mean state exhibits a high 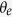 and precipitation over the Indian Ocean, Indian Peninsula, and along the southern slopes of the TIP, and minimum precipitation over the central Arabian Peninsula (Fig. 3c). This basic pattern in the observations is reasonably simulated in the CON experiment (Fig. 3g). However, in the NoTIP run over North India, the surface 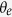 weakens remarkably and the monsoon rainfall disappears (Fig. 3k). Is this due to the removal of the TIP such that the cold/dry advection can destroy the north branch of the SASM? If this were true, then replacing the TIP barrier would recover the observed mean state. To test this, a third experiment, TIP_NS, is designed, which is also the same as CON except that the surface sensible heating of the TIP region is not allowed to heat the atmosphere, so that the TIP merely acts as a barrier to block the tropics from the subtropics. The results shown in Fig. 4c are similar to those in the NoTIP run (Fig. 3k): over North India the simulated 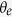 is still low and the monsoonal precipitation does not recover. In the subtropical continental area the surface potential temperature in the TIP-NS experiment is even weaker compared to the NoTIP experiment because in the NoTIP run the surface sensible heating over the TIP platform, which is 500 m above sea level, remains, indicating the importance of TIP thermal impacts. Actually, the mean surface air temperatures averaged from the four grid points as denoted in Fig. 3c,g,k, which are located over northeastern India and associated with the maximum monsoon precipitation in the reanalysis (Fig. 3c), are all around 30 °C in ERA40 and in all experiments (bottom row of Fig. 3), indicating again that cold advection does not occur in the region even if the TIP is removed. The above results thus imply that a significant decrease of 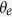 in this region in either NoTIP or TIP_NS (Figs 3k and 4c) must be due mainly to the reduced water vapor content (Figs 3j and 4b).

## Hydrological Perspective: water vapor transport from the sea is crucial for the continental monsoon

Figures 3 and 4 present the relative contributions of 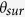 (top row) and *q*_*sur*_ (second row) to 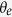 (third row) in ERA40 and all experiments. The distribution of 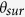 is relatively uniform except over mountainous regions, and it is warmer over land than over the ocean, while the distribution of *q*_*sur*_ resembles that of 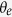 in all cases. In ERA40 and CON (Fig. 3a,b,e,f) except over the TIP, the high 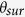 is located over the Arabian Peninsula, while the *q*_*sur*_ maxima are located over the oceans and Northeast India. In the NoTIP run, the distribution of 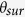 is more uniform in the subtropics (Fig. 3i), while *q*_*sur*_ decreases remarkably over Northeast India (Fig. 3j), similar to the decrease of 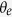 in the same region (Fig. 3k). The distributions of 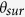, *q*_*sur*_, and 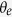 in the TIP_NS experiment (Fig. 4a–c) resemble, respectively, those in the NoTIP experiment (Fig. 3i–k).

The lifting condensation level (LCL) is a measure of the water vapor content in the lower troposphere: the lower the LCL, the higher the water vapor content. In the bottom row of Fig. 3 are shown the LCL distributions over Northeast India. The LCL is low (at 850 hPa) in ERA40 and the CON run, but high (above 700 hPa) in NoTIP, indicating that the water vapor content is much lower in NoTIP than in ERA40 and CON. The Convective Available Potential Energy (CAPE), which is measured by calculating the temperature difference between a rising air parcel (blue dashed) and the environment (red solid), is also shown in the figure. ERA40 shows active convection in the North India region, with a CAPE of 796.41 J kg^−1^ (Fig. 3d). This feature is well simulated in CON, with a stronger CAPE of 1616.31 J kg^−1^ (Fig. 3h). In NoTIP, however, the potential convection diminishes because the temperature of the air parcel is colder than the environment at all levels, and the CAPE disappears (Fig. 3l). We then infer that in NoTIP, although 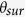 over North India does not change compared to 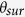 in ERA40 and CON, *q*_*sur*_ becomes much lower, causing the reduced surface energy and suppressed rainfall there (Fig. 3k).

The difference between CON and TIP_NS can to a certain extent present the thermal impacts of the TIP-SHAP. The 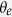 difference (Fig. 4f) is significantly positive over North India and East Asia, accompanied by a remarkable increase in precipitation. The difference in 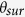 is mainly limited to over the two mountainous regions (Fig. 4d) and is insignificant over North India, while the difference in *q*_*sur*_is remarkably positive over North India and East Asia (Fig. 4e), as is the 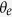 difference (Fig. 4f).

It is important to note the similarity in the distributions of *q*_*sur*_ and 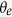 (Fig. 4e,f). Actually, from (1) the dependence of the relative change in surface 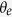 on the changes in 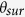 and *q*_*sur*_ can be evaluated as:


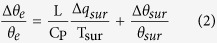


or,


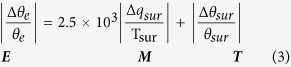


Where “|a|” indicates a nondimensional variable “a”. The distributions of the three terms (***T, M***,and ***E***) in (3) for the difference between CON and TIP_NS are presented, respectively, in Fig. 4g–i. It becomes evident that except over the mountainous regions, the *q*_*sur*_ term (Fig. 4h) prevails over the 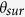 term (Fig. 4g) in determining the change in surface entropy (Fig. 4i) over North India and East Asia.

Local water vapor content is influenced by the convergence of water vapor transport. In ERA40 and the CON run, the 850-hPa water-vapor conveyer belt in the tropics deviates northward toward North India and the southern slope of the TP (Fig. 3b,f), resulting in the high *q*_*sur*_ there. In the NoTIP and TIP_NS runs, such northward water vapor transport is remarkably reduced, resulting in the low *q*_*sur*_ over North India (Figs 3j and 4b). Consequently, continental rainfall is suppressed (Figs 3k and 4c), and the northern branch of the SASM disappears. In the CON-TIP_NS case, the increased *q*_*sur*_ over the continent is also accompanied by much stronger water vapor transport from the Indian Ocean to the tropical and subtropical Asian continent (Fig. 4e).

**Figure 5:**
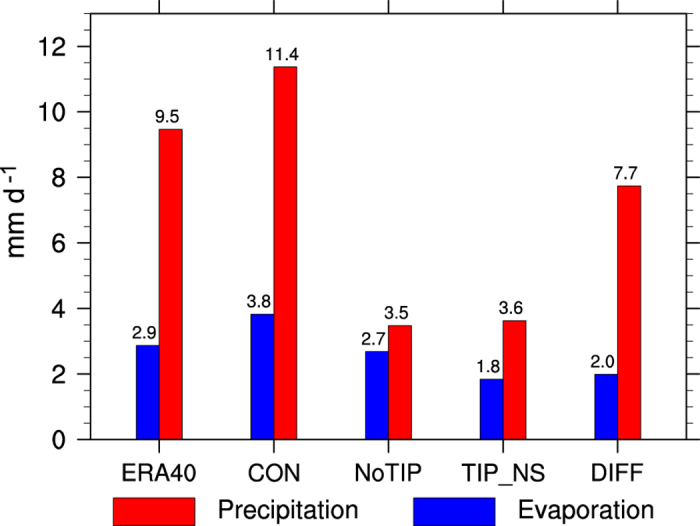
Hydrological budgets (mm d^−1^) in the SASM region of (24–28°N, 75–100°E) for ERA-40 and experiments CON, NoTIP, and TIP_NS. DIFF denotes the difference (CON-TIP_NS). This figure was generated using NCL version 6.3.0: http://dx.doi.org/10.5065/D6WD3XH5.

Local surface evaporation is another source of atmospheric water vapor. To evaluate its relative contribution to the monsoon rainfall, we calculated the regional means of evaporation and precipitation over the boxed region (24–28°N, 75–100°E) shown in Figs 3 and 4, which cover North India and the southern slope of the Himalayas. Results demonstrate that local evaporation is much less than precipitation (Fig. 5) both in ERA40 (2.9 versus 9.5 mm d^−1^) and in CON (3.8 versus 11.4 mm d^−1^). This means that even if all the surface water vapor that evaporated from the ground were condensed and recycled locally, it would account for only about one-third of the local precipitation, not to mention that the precipitation recycling ratio is far below 0.5 in the Indian area^26^. In both the NoTIP and TIP_NS runs, the local evaporation does not decrease much, but the precipitation decreases substantially, from 11.4 mm d^−1^ in CON to 3.5 mm d^−1^ in NoTIP and 3.6 mm d^−1^ in TIP_NS. The results of CON minus TIP_NS show that the thermal impact of the TIP can increase evaporation by merely 2.0 mm d^−1^, but it can increase precipitation by 7.7 mm d^−1^, which accounts for more than two-thirds of the total precipitation in CON. All these results indicate that local evaporation does not contribute significantly to the successive strong precipitation of the ASM, while the water vapor transport from outside does. It can be inferred that water vapor advection from the Indian Ocean induced by the TIP-SHAP can make a substantial contribution to producing high entropy and convection over North India and East Asia and forming the continental monsoon.

## Dynamics of horizontal water vapor transport: thermal pumping of the TIP

**Figure 6:**
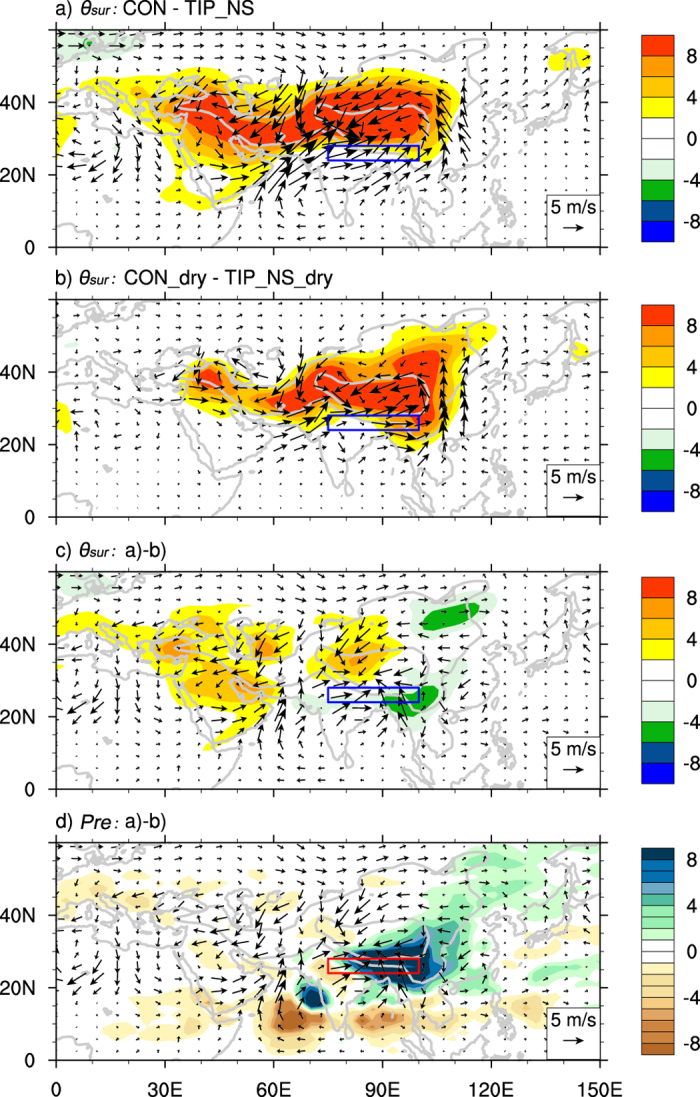
JJA-mean differences of *θ*_*sur*_ (shading, K) and surface circulation (vectors, m s^−1^) between (a) CON and TIP_NS, (b) CON_dry and TIP_NS_dry, (c) difference between (a) and (b); (d) same as (c) but for precipitation (mm d^−1^). The square indicates the SASM region of (24–28°N, 75–100°E). This figure was generated using NCL version 6.3.0: http://dx.doi.org/10.5065/D6WD3XH5.

Why can the TIP-SHAP transport water vapor horizontally from sea to land? According to the 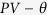 theory^27^,^28^,^29^, a warm surface potential temperature 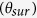 anomaly can generate a positive potential vorticity (*PV*) and cyclonic circulation near the surface. The difference between the CON and TIP_NS runs (Fig. 6a) shows that the surface sensible heating over the TIP can result in a positive 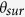 anomaly, with a maximum of 8K over the TIP. Accordingly, two surface cyclonic circulations are generated surrounding the IP and TP, respectively, forming an enhanced strong southwesterly over South Asia and a southerly over East Asia and leading to the effective water vapor transport from sea to land. These results can be well interpreted from the above 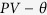 perspective. It is this fundamental response of 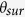 to the surface sensible heating over the TIP (TIP-SHAP) and the resultant atmospheric circulation that generate the high surface entropy and rain belt over North India, the southern slope of the TP, and East Asia, forming the northern branch of the SASM as well as the East Asian monsoon.

To evaluate the dependence of the TIP-SHAP on moist processes, we carry out two more experiments, CON_dry and TIP_NS_dry, under dry conditions, which are reproduced respectively from the CON and TIP_NS experiments except that the water vapor in the atmosphere is set to zero so that moist convection is excluded (Methods). The differences in 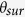 and the surface circulation between CON_dry and TIP_NS_dry are shown in Fig. 6b. The 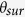 difference is positive, with a similar intensity of 8K, but it is more confined to the plateau area; and the surface cyclonic circulation and the onshore wind induced by the TIP-SHAP in dry conditions are, although weaker, similar to those under moist conditions (Fig. 6a), demonstrating that the initiation of the TIP-SHAP does not rely on moist processes.

**Figure 7:**
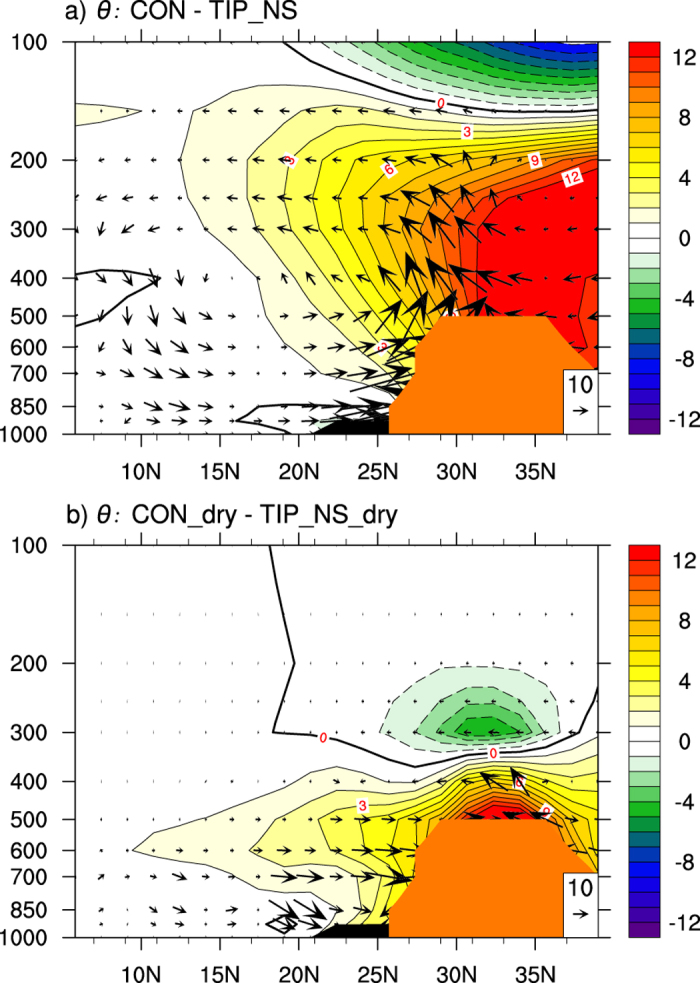
JJA-mean differences along 90°E of *θ* (K) and circulation (vectors, *v* in m s^−1^, –*ω* has been amplified by 300 and is in hPa s^−1^) of (a) CON-TIP_NS, and (b)CON_dry-TIP_NS_dry. This figure was generated using NCL version 6.3.0: http://dx.doi.org/10.5065/D6WD3XH5.

However, moist processes have a strong positive feedback on the general circulation of the atmosphere. The depth of the positive 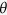 difference between the runs with and without surface sensible heating over the TIP extends to above 200 hPa in the moist experiment (Fig. 7a) but is quite shallow in the dry experiment (Fig. 7b). The differences in 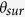 and surface circulation between Fig. 6a,b as shown in Fig. 6c present the impact of moist processes on the pumping effect of the TIP-SHAP: they intensify the cyclonic circulation surrounding the TIP with a remarkable convergence over its southeast and produce increased 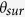 to the west of 90 oE and decreased 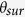 to the east and over North India. This 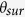 difference coincides well with the precipitation difference (Fig. 6d): the area with increased rainfall corresponds to reduced surface 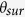 and vice versa, presenting a negative feedback of monsoon rainfall on surface energy, though it is secondary.

## Summary and Discussion

Based on astronomical radiation analysis, reanalysis data diagnosis, and numerical experiments, we have demonstrated that in winter the Tibetan-Iranian Plateau cannot shield India from cold/dry northerly intrusion since the frequent cold/dry northwesterlies or northeasterlies can go around the giant TIP by surrounding its southern rim intruding into India. In summer, on the other hand, in the South Asian summer monsoon sector the midlatitudes and subtropics receive more solar energy and possess higher surface potential temperature than North India, and there is no cold/dry advection from the north into the SASM area so that TIP shielding does not exist. Results from the hydrological analysis demonstrate that over North India local evaporation can account for only a small portion of precipitation, and the change in surface entropy is essentially attributed to the change in water vapor. The 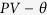 diagnosis reveals that the cyclonic circulation surrounding the TIP in the lower layers and the remarkable onshore wind along 20 °N are the atmospheric response to the thermal forcing of the Tibetan-Iranian-Plateau sensible-heat-driven air-pump (TIP-SHAP); and its generation does not rely on moist processes, although these processes have a significant positive feedback on the cyclonic circulation. The TIP-SHAP–induced cyclonic circulation transports enormous quantities of water vapor from the oceans to the far north Indian Peninsula, the foothills of the Himalayas, and East Asia, leading to high surface entropy and strong and persistent precipitation there. Therefore the TIP-SHAP has a dominant influence on moist convection and the continental summer monsoon over North India and East Asia.

Results from this study imply that protecting the ecosystems of the TIP and its thermal status can not only improve the local environment, but can also influence the global climate, particularly the Asian monsoon.

## Methods

### Graphic software

All figures were produced by NCAR Command Language (NCL) version 6.3.0, open source software free to public, by UCAR/NCAR/CISL/TDD, http://dx.doi.org/10.5065/D6WD3XH5, while Fig. 1a was additionally modified by licensed Microsoft PowerPoint and Illustrator. Table 1 was created by the licensed Microsoft Excel.

### Data Sources

The climate incoming Daily Solar Radiation (DSR) used in Fig. 1b is from Clouds and the Earth’s Radiant Energy System (CERES)^23^ Level3B datasets at http://ceres.larc.nasa.gov/index.php.

The DSR for 2001 used in Fig. 1c is from CERES level3 datasets.

The daily near-surface wind and air temperature for 2001 used in Fig. 1d are from the European Centre for Medium-Range Weather Forecasts (ECMWF) ERA-interim^24^ dataset at http://apps.ecmwf.int/datasets/data/interim-full-daily.

The monthly mean reanalysis dataset used in Figs 2, 3, 4, 5 is from ERA-40^25^ between 1958 and 2002 at http://apps.ecmwf.int/datasets.

All of the variables are interpolated from pressure coordinates to sigma coordinates at the σ = 0.99 level near the surface.

The precipitation dataset used in Fig. 3c is from the Global Precipitation Climatology Project (GPCP)^30^ monthly mean data from 1979 to the present at http://www.esrl.noaa.gov/psd/data/gridded/data.gpcp.html.

### Calculation of the Daily Solar Radiation (DSR) at Summer Solstice

At the summer solstice, the solar declination angle *δ* = 23.5°N. The daily sunshine durations at latitude A 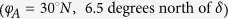 and B 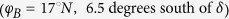 can be calculated as shown below.

The relation between the solar zenith angle 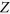 and the hour angle 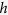 satisfies:


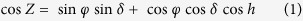


At sunrise 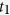 and sunset 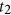 *Z* equals 90°. Therefore the hour angles *h*_*A*_ and *h*_*B*_ at sunrise and sunset for latitudes A and B are estimated by:


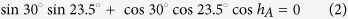



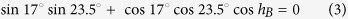



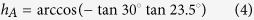



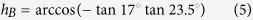


Consequently the sunshine duration (daytime) is 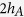 (unit: radians) at *ϕ*_*A*_ and 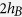 at *ϕ*_*B*_. For the units in hours, the sunshine duration becomes:


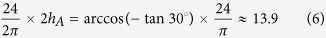



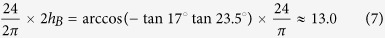


(6) and (7) suggest that the sunshine duration at 30 °N is about one hour longer than at 17 °N. Meanwhile, the total shortwave radiation at the top-of-atmosphere (TOA) in an integral for daytime is:


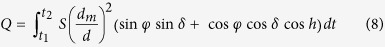


Where 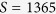 W m^−2^ and denotes the solar constant, 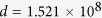 m and denotes the distance between the sun and the earth, 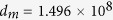 m and denotes its mean value, and 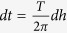 with *T* being 86400 seconds. Then


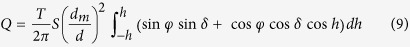


Therefore the total daily solar radiation (DSR) at latitudes A and B is, respectively:


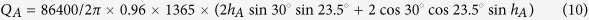



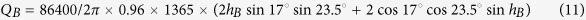


From (10) and (11), 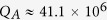 J m^−2^ and 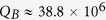 J m^−2^, respectively, which is identical to 476 W m^−2^ at 30 °N and 448.9 W m^−2^ at 17 °N for the DSR at the TOA.

### Model introduction and experiment design

The model used in this study is the Spectral Atmospheric Model of LASG/IAP (known as SAMIL)^31^,^32^,^33^, which has the horizontal resolution R42 (2.81° longitude × 1.66° latitude) with 26 vertical layers in σ–p hybrid coordinates, extending from the surface to 2.19 hPa. The mass flux cumulus parameterization of Tiedtke (1989)^34^ is used to calculate convective precipitation. The cloud scheme is a diagnostic method parameterized by low-layer static stability and relative humidity^35^,^36^. A stratocumulus scheme is also employed, based on a statistical cloud scheme^37^. A nonlocal scheme is employed to calculate the eddy-diffusivity profile and turbulent velocity scale, and the model incorporates nonlocal transport effects for heat and moisture^38^. The radiation scheme employed is the Edwards–Slingo scheme^39^, but with some improvement by Sun^40^,^41^. SAMIL is coupled with the land model NCAR CLM3^42^ in this study^33^.

Five experiments are carried out in this study. The detailed experimental design is shown in Table 1. The CON run is forced by the observed monthly mean sea surface temperature (SST)^43^ averaged between 1990 and 1999 and the realistic topography. The NoTIP run is the same as the CON except the Tibetan-Iranian Plateau (TIP) is removed where the topography is above 500 m. The TIP_NS run is also the same as the CON but the vertical diffusion heating in the atmosphere is set to zero over the TIP region where the elevation is above 500 m during the integration. CON_dry and TIP_NS_dry are, respectively, the same as CON and TIP_NS except the water vapor in the atmosphere is set to zero during the integration in both experiments. All of the experiments are integrated for 10 years, and the mean of the last 5 years is calculated as the equilibrium state.

## Additional Information

**How to cite this article**: He, B. *et al.* Astronomical and Hydrological Perspective of Mountain Impacts on the Asian Summer Monsoon. *Sci. Rep.* **5**, 17586; doi: 10.1038/srep17586 (2015).

## Figures and Tables

**Table 1:** Experiment design.

Experiments	TIP topography	TIP Sensible heating	Moist process
CON	Yes	Yes	Yes
NoTIP	No	Yes	Yes
TIP_NS	Yes	No	Yes
CON_dry	Yes	Yes	No
TIP_NS_dry	Yes	No	No
